# Supplementary material for: Single particle cryo-EM structure of the outer hair cell motor protein prestin
Source: Nat Commun. 2022 Jan 12;13:290. doi: 10.1038/s41467-021-27915-z (PMC8755724; doi:10.1038/s41467-021-27915-z)
Supplement: Supplementary file 1 — Supplementary Information [file 41467_2021_27915_MOESM1_ESM.pdf]

Single particle cryo-EM structure of the outer hair cell motor protein prestin

Carmen Butan, Qiang Song, Jun-Ping Bai, Winston J. T. Tan,
Dhasakumar Navaratnam, Joseph Santos-Sacchi

Supplementary Figures

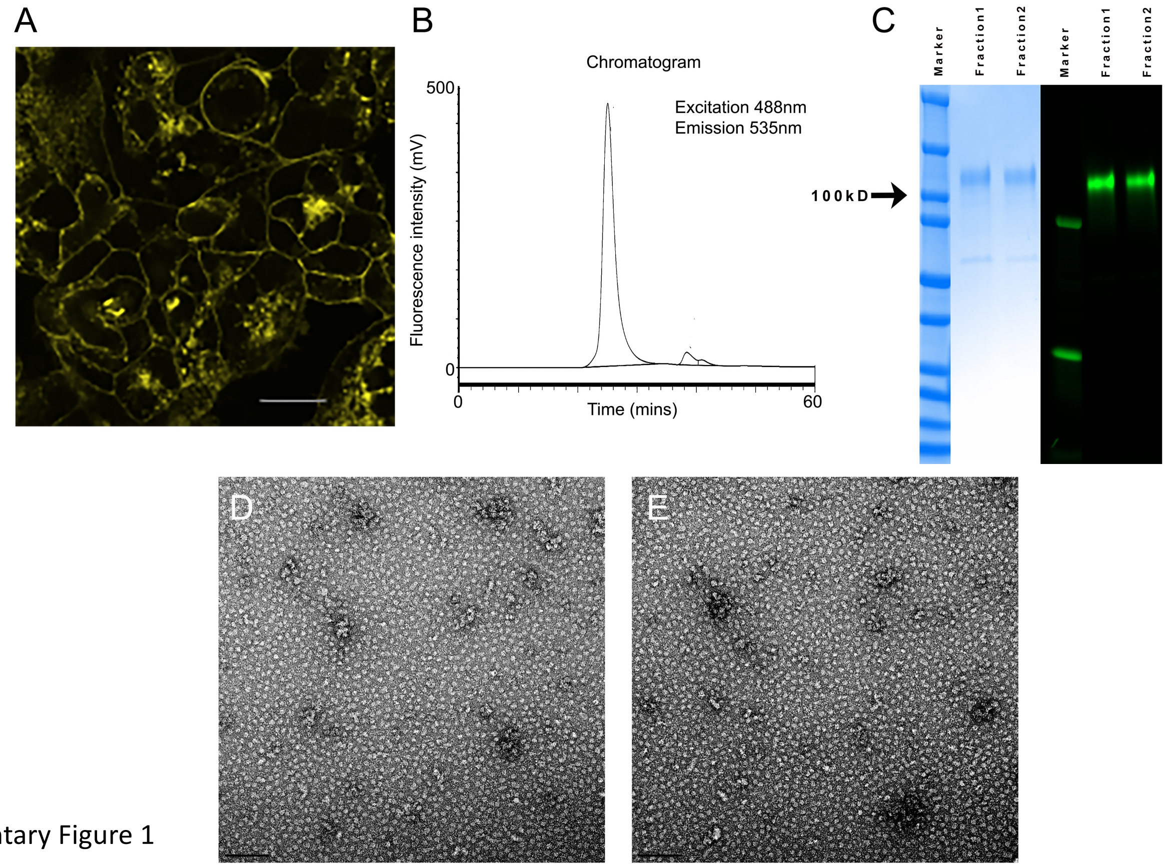

Supplementary Figure 1

Supplementary Figure 1. (**A**). The figure shows a fluorescence image of HEK 293 cells 48 hours after tetracycline induction with prestin YFP expressed in the plasma membrane of cells. Scale bar is 10 microns. (**B**) Fluorescence Size Exclusion Chromatogram (FSEC) of purified prestin YFP injected into a Superdex 200 Increase 10/300 GL Column. Two fractions corresponding to the single monodisperse peak were collected and used for cryo-EM analysis. (**C**). Coomasie blue staining (left three lanes) and fluorescence in gel imaging of the two fractions obtained from FSEC confirmed the presence of purified prestin-YFP (~110 kDa). The position of the 100 kDa molecular weight marker is indicated. (**D, E**). Representative negative stain micrographs of fractions collected at the peak of the SEC profile of prestin purified in digitonin/GDN. Fractions from the peak from more than 5 separate experiments of protein expression and purification were used to optimize the cryo-grids for determining the 3.6 Å, high-resolution structure of prestin. Scale bars are 100 nm. Source data are provided as a Source Data file.

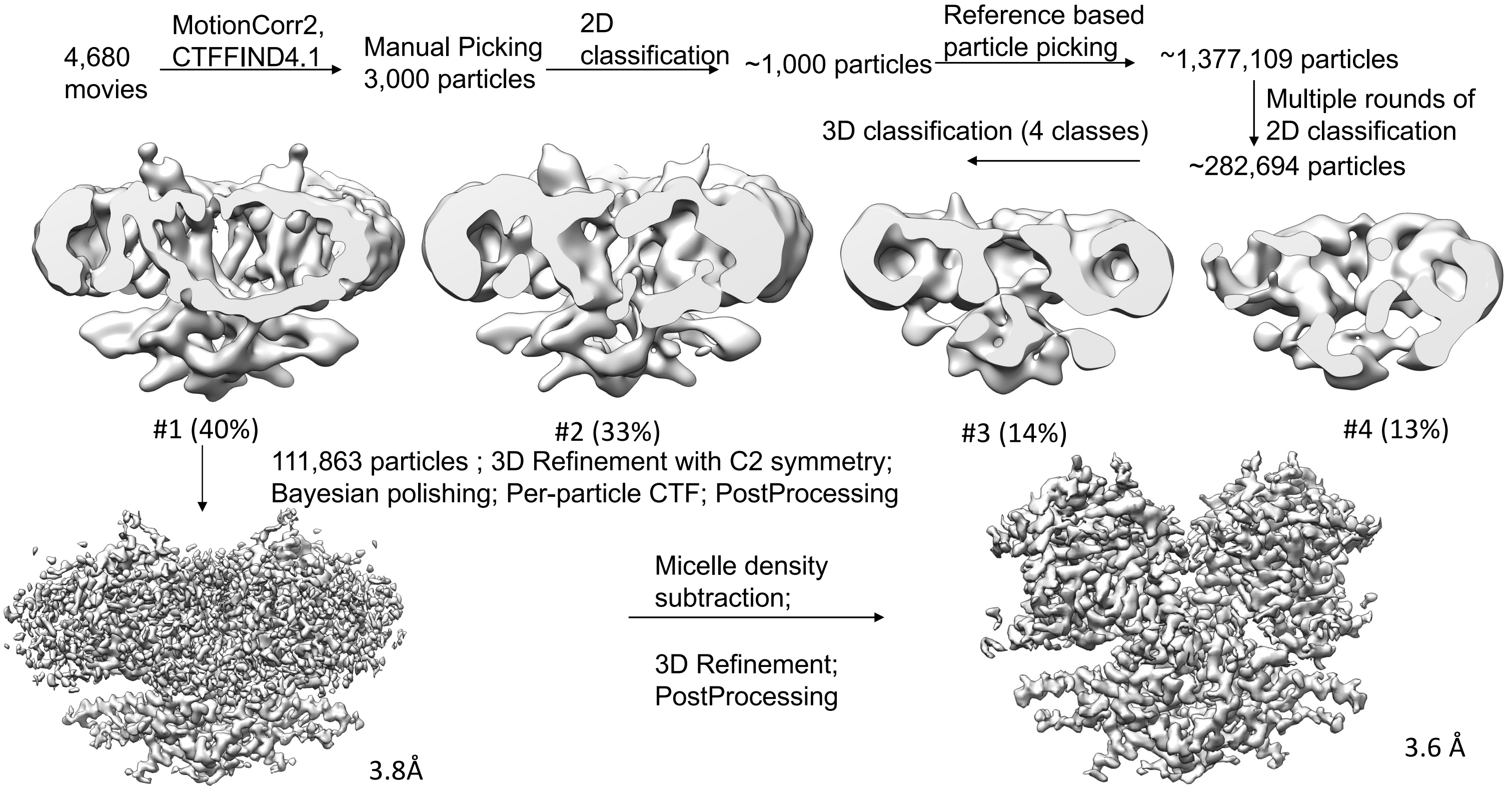

Supplementary Figure 2

Supplementary Figure 2. The workflow of the cryo-EM image processing. 1,377,109 extracted particles were subjected to multiple rounds of 2D classification at the end of which 282,694 particles were selected. These particles were subjected to 3D classification in 4 classes without symmetry and the particles in class #1 (40%) were further processed with two-fold symmetry generating a 3.6 Å resolution cryo-EM map.

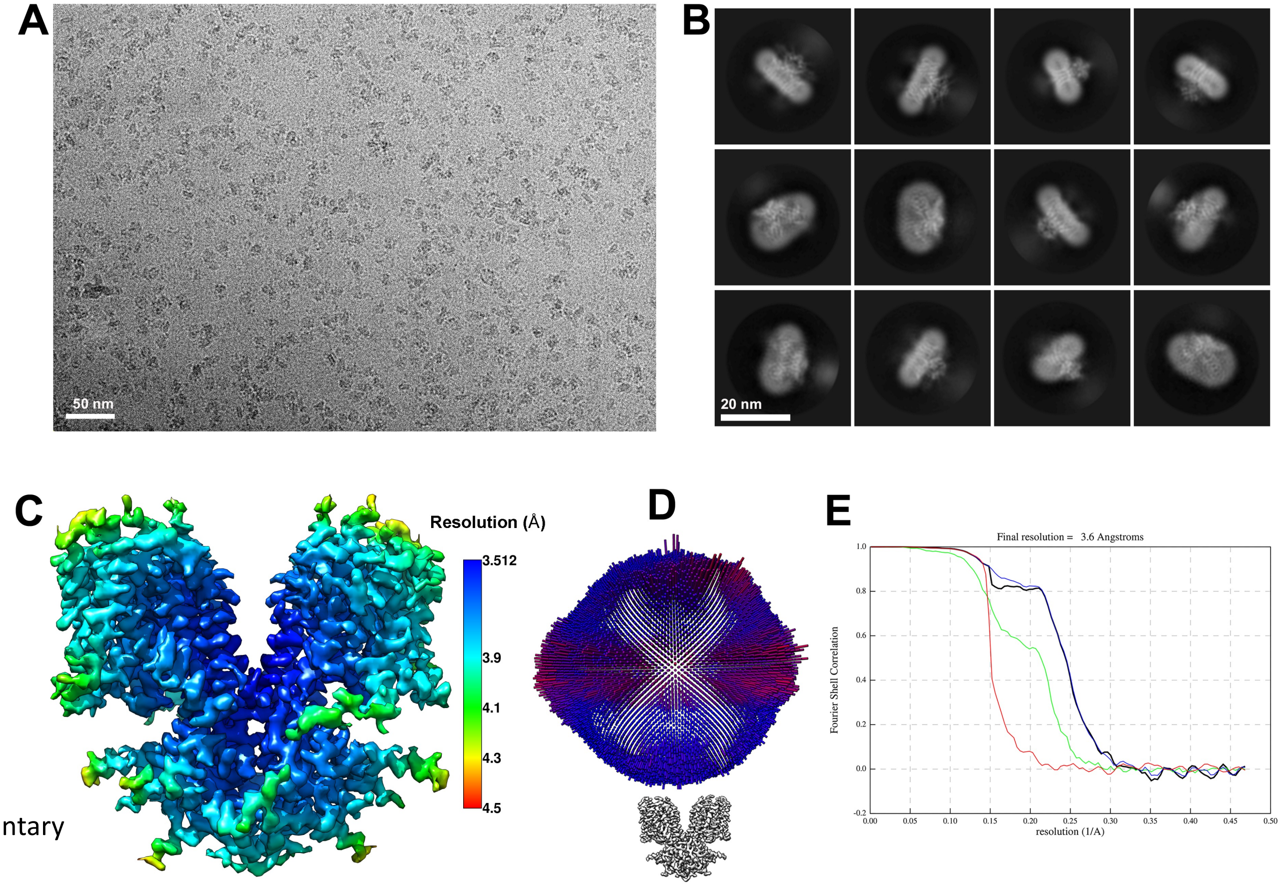

Supplementary Figure 3

Supplementary Figure 3. Cryo-EM imaging of prestin. (**A**) Representative cryo-EM micrograph of fractions collected at the peak of the SEC profile of prestin purified in digitonin/GDN. Fractions from the peak from more than 5 separate experiments of protein expression and purification were used to optimize the cryo-grids for determining the 3.6 Å, high-resolution structure of prestin; (**B**) Selected reference-free 2D class averages; (**C**) Cryo-EM density map of prestin colored according to local resolution. (**D**) Angular distribution of the particle images used for calculating the final cryo-EM map (111,863 particles). The length and color of cylinders (red and blue) is proportional to the number of particles visualized for that particular orientation. (**E**) Gold standard FSC curves from RELION indicate that the cryo-EM structure of prestin has a nominal resolution of 3.6 Å at FSC=0.143.

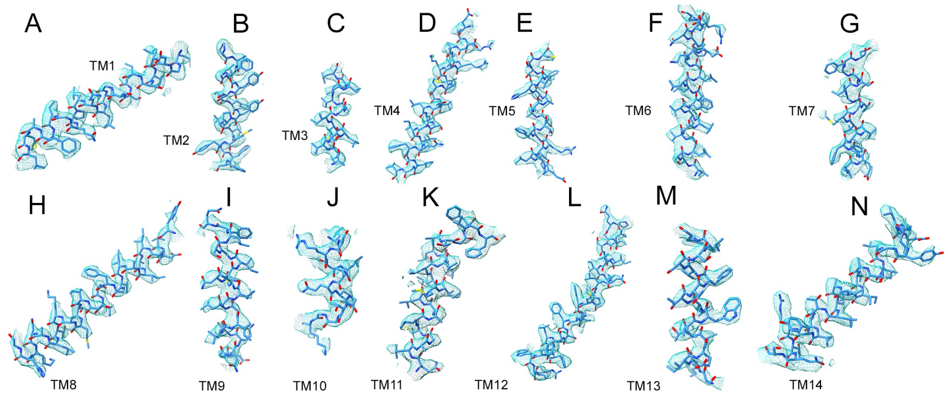

Supplementary Figure 4

Supplementary Figure 4. Representative densities for the transmembrane regions. (**A** to **N**) Cryo-EM densities with the atomic models for individual transmembrane helices.

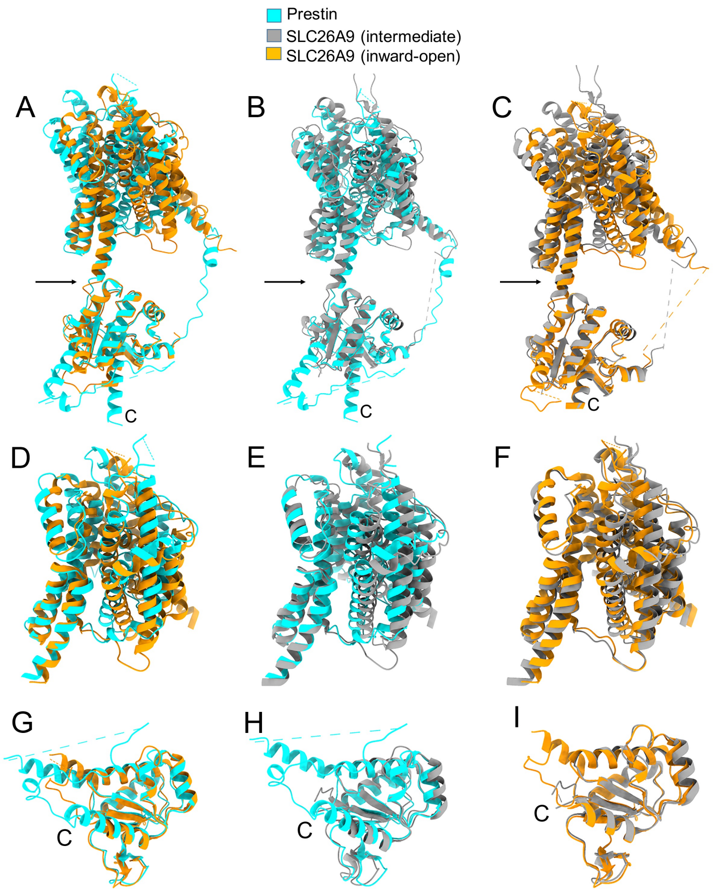

Supplementary Figure 5

Supplementary Figure 5. Structural comparison of a single monomer of prestin and Slc26a9 structures. (**A,B,C**) Superposition of the monomeric structures of prestin (cyan) and Slc26a9 (in two distinct states: orange in inward-open and gray in intermediate). The monomers were superposed through a segment within the STAS domain (using residues Gln504-Ala578 of the prestin structure and the corresponding residues of the Slc26a9 structures). Arrows indicate a possible hinge at the connection between the cytosolic and transmembrane domains. (**D,E,F**) Superposition of the separated transmembrane domain of prestin with the separated transmembrane domains of Slc26a9 in “inward-open” and “intermediate” conformations. (**G,H,I**) superposition of the separated STAS domain of prestin with the separated STAS domains of Slc26a9 in “inward-open” and “intermediate” conformations. C, C terminus.

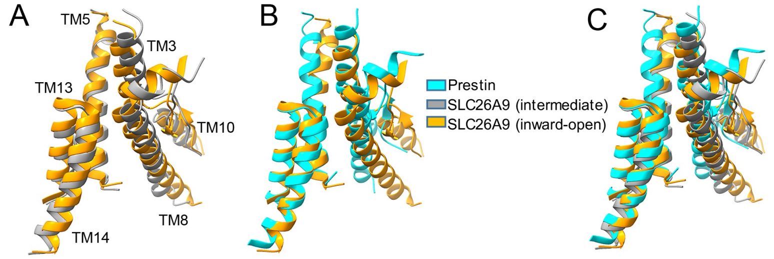

Supplementary Figure 6

Supplementary Figure 6. Conformational differences between the transmembrane domains of one prestin subunit and one Slc26a9 subunit. (**A**) Close-up view of the (TM3, TM5, TM8, TM10, TM13 and TM14) helices from the superposition of the transmembrane domains of Slc26a9 in two distinct states (PDB 7CH1, in orange ribbon and PDB 6RTF, in grey ribbon). The TM13 and TM14 helices of the "inward-open" Slc26a9 structure (PDB 7CH1) were used as a reference for a match with the corresponding region in the intermediate Slc26a9 structure (PDB 6RTF). (B) Close-up view of the (TM3, TM5, TM8, TM10, TM13 and TM14) helices from the superposition of the transmembrane domain of prestin (cyan) and the corresponding region of Slc26a9 (PDB 7CH1, in orange ribbon), which was the reference structure. The structural alignment was restricted to the transmembrane 13 and 14 helices. (C) Structural comparison of the three overlaid structures (prestin and SLc26a9 in two different states) focusing on the (TM3, TM5, TM8, TM10, TM13 and TM14) helices.

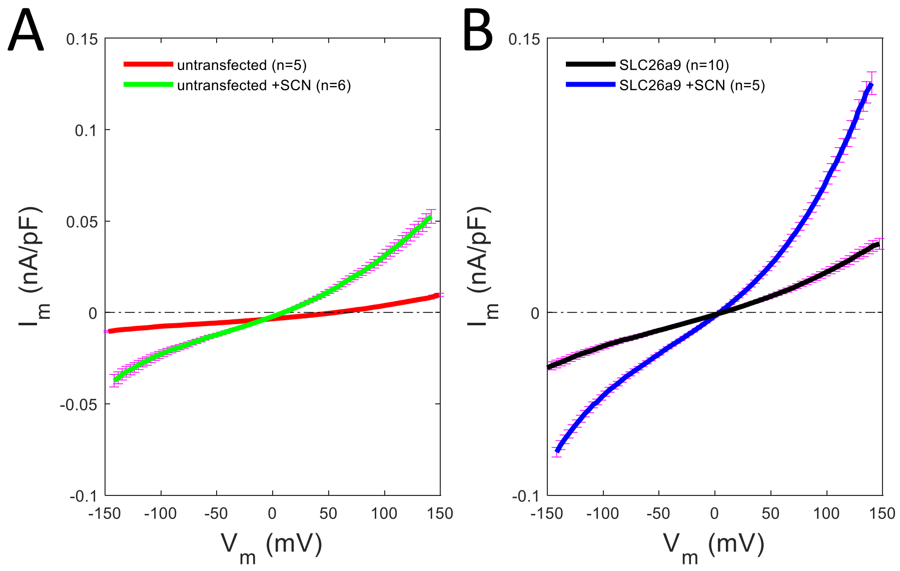

Supplementary Figure 7

Supplementary Figure 7. (**A**) Effect of SCN^- on the magnitude of ramp induced current in un-transfected CHO cells. A slight increase in current is observed with SCN^- (green) compared to Cl^- (red). The data are presented as means (+/- SEM) of currents. N=5 for Cl^-, N= 6 for SCN^-. (**B**) In Slc26a9 transfected cells, larger currents compared to untransfected cells are observed without (Cl^-, black) and with SCN^- (blue), indicating successful delivery of the protein to the membrane. The data are presented as means (+/- SEM) of currents. N=10 for Cl^-, N= 5 for SCN^-. Our maximum average current in the absence of SCN^- (that is, in the presence of Cl^-) for untransfected cells is 0.01 nA/pF and 3.7 fold higher at 0.037 nA/pF for Slc26a9 transfected cells. This is comparable to the values in Walter et al (2018). In the presence of SCN^-, it is an additional 3.5 fold higher at 0.13 nA/pF. Source data are provided as a Source Data file.

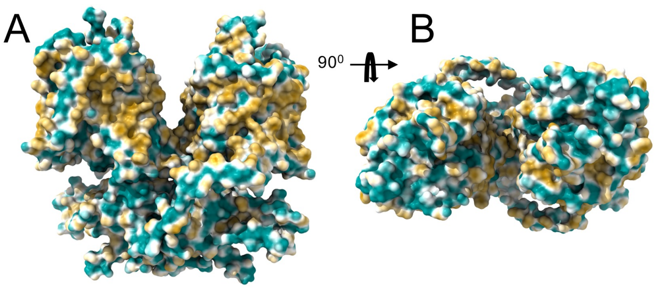

Supplementary Figure 8

Supplementary Figure 8. Representation of hydrophobicity on the molecular surface of prestin with the most extensive hydrophobic patches located within the TM domains of the protein (in sepia). Hydrophilic residues are shown in turquoise and hydrophobic residues are shown in sepia. (**A**) Side view of the hydrophobicity mapping. (**B**) Extracellular view of the hydrophobicity mapping.

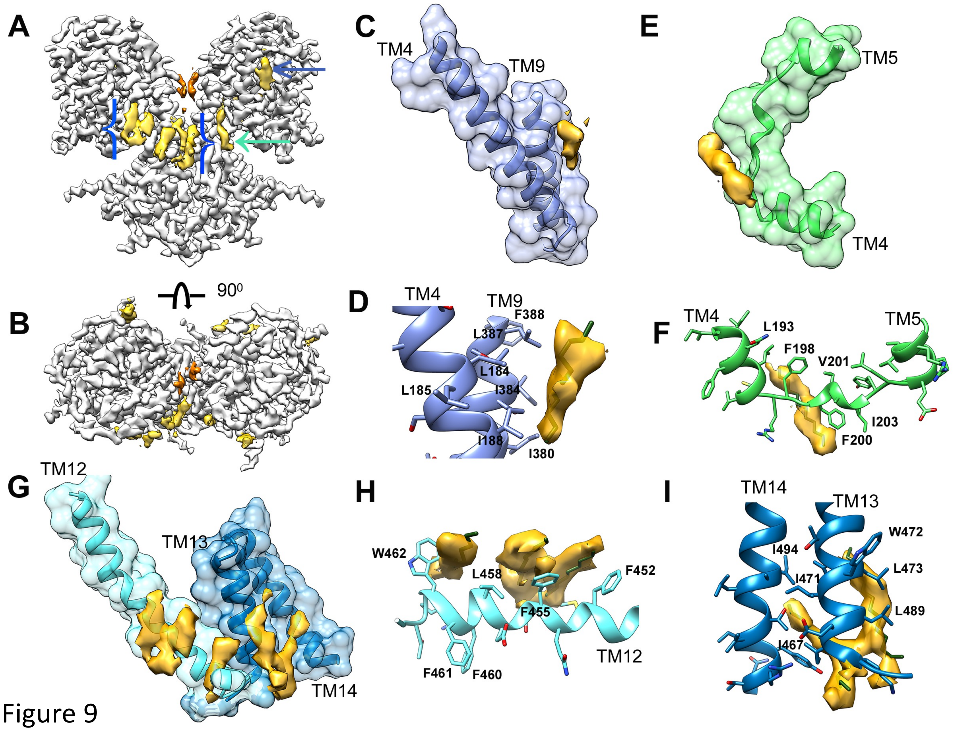

Supplementary Figure 9

Supplementary Figure 9. Protein lipid interactions. (**A,B**) The prestin surface is decorated with densities which might be assigned to stably bound membrane lipids or micelle molecules. In particular, the space between the transmembrane domains is filled by amorphous, non-protein densities when the map's contour level is increased (some putative lipids occupying this space are shown in orange). Interestingly, when the cryo-EM structure of the human prestin in the contracted state (PDB 7GLU) is overlaid onto our cryo-EM map of prestin, several resolved lipid molecules fit reasonably well into the "unassigned for densities" decorating our prestin structure. "Unassigned for densities" are shown in gold. (**C,D**) Details on the positioning of the gold density (identified by a lavender arrow in panel A) relative to the prestin surface. It "decorates" TM4 and TM9 of the gerbil prestin structure. (**D**) Several hydrophobic residues, shown in stick representation (L184, L185, I380, I384, L387, F388) are pointing towards the interface with the putative lipid density. (**E,F**) The density pointed at by a green arrow in panel A interfaces with the loop connecting the transmembrane helices 4 and 5 and it is located close to the separation between the "gate" and "the core" domain at the cytosolic entry path. (**F**) A cluster of hydrophobic residues shown in stick representation (L193, F198, F200, V201, I203) are exposed towards the putative lipid density. (**G,H,I**) The gold density patch identified by cyan brackets in panel A interfaces with (TM12, TM13 and TM14). (**G**) Enlarged view showing the positioning of the gold densities relative to the prestin surface. (**H, I**) Close-up view of side chains facing towards the patch of putative lipid densities.

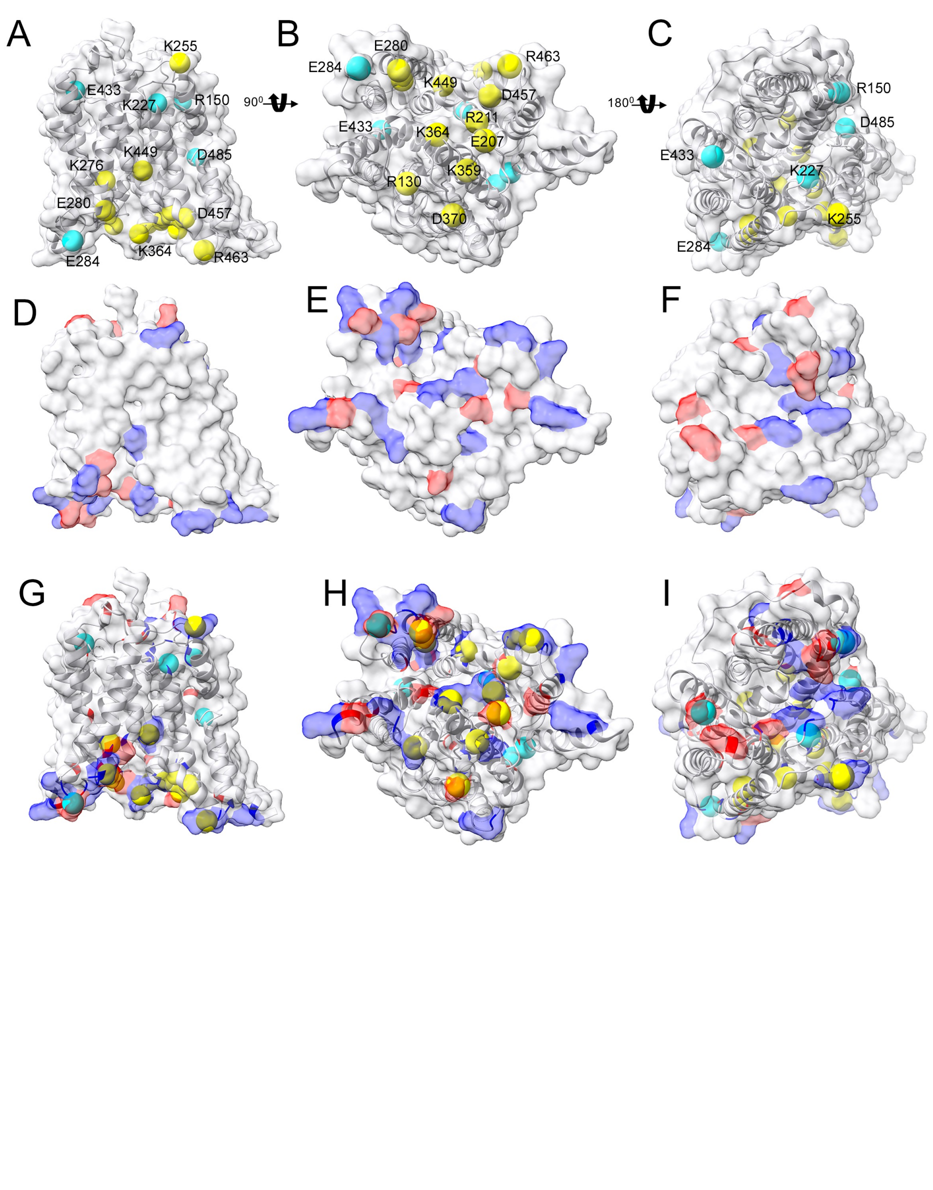

Supplementary Figure 10

Supplementary Figure 10. Mapping of charged residues important for the nonlinear capacitance onto the transmembrane structure of a prestin subunit. (**A, B, C**) Three views of the transmembrane domain of a prestin subunit shown in transparent surface representation. The position of charged residues (R130, E207, R211, K255, K276, E280, K359, K364, D370, K449, D457, R463) which are important for the NLC is highlighted by yellow spheres. Mapping of these functionally important residues onto the prestin surface shows that they cluster preferentially within the cytosolic exposed surface. Positions of charged residues (R150, K227, E284, E433, D485) which are not important for the NLC are highlighted by cyan spheres. (**D,E,F**) Molecular surface of the transmembrane region of a prestin subunit shown in analogous views to (**A,B,C**) and color-coded in blue for positively charged residues (Arg and Lys) and color-coded in red for negatively charged residues (Glu and Asp). (**G, H, I**) Molecular surface of the transmembrane region of a prestin subunit colored coded for positively (Arg and Lys) and negatively charged residues (Glu and Asp) indicating the residues which are important (yellow spheres) or not important (cyan spheres) for NLC. The views are analogous to those in (**D,E,F**).

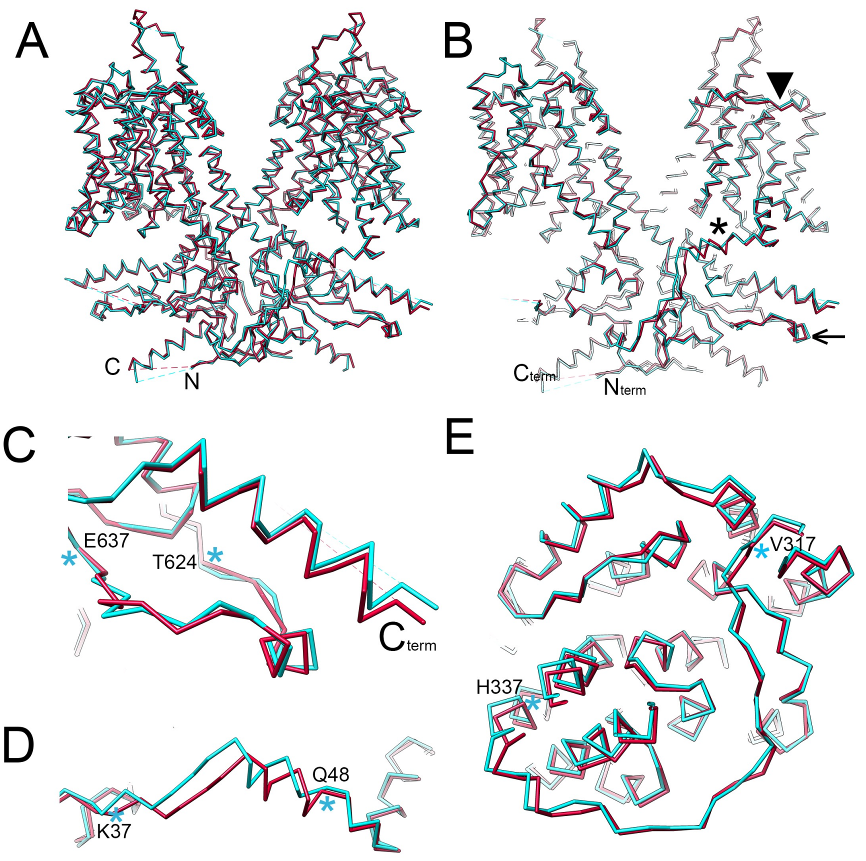

Supplementary Figure 11

Supplementary Figure 11. Structural comparison of the gerbil prestin structure to the human prestin structure in the contracted state(7LGU). (**A**) Overlay of gerbil prestin (in cyan) and human prestin (in dark red) indicates highly similar structures. (**B**) The greatest differences between the two structures occur at regions highlighted by an arrow, an arrow head, and an asterisk. (**C,D,E**) The differences include regions in the STAS domain (residues 624-637) (close-up view in panel C and indicated by an arrow in panel B), in the transmembrane domain (residues 317-337, which is the loop connecting TM7 and TM8) (close-up view in panel E and indicated by an arrowhead in panel B) and especially residues (37-48) at the cytosolic N-terminus (close-up view in panel D and indicated by an asterisk in panel B).

**Data collection and Statistics of cryo-EM data collection and structure determination**	
**Image processing**	
Microscope	Titan Krios
Voltage (KV)	300
Camera	Gatan K3
Magnification	81,000
Electron exposure (e^-/Å^2)	54
Defocus range (μm)	1.15-2.15
Pixel size (Å)	0.534
Symmetry imposed	C2
Micrographs	4,680
Final particle images	111,863
Map resolution (Å)	3.6
Map resolution threshold (FSC)	0.143
Map sharpening B factor (Å ^{2})	-128
**Model Refinment**	
R.m.s deviation bonds (Å)	0.003
R.m.s deviation angles (0)	0.68
**Validation**	
MolProbity score	1.64
Clash score	7.39
Rotamer outliers (%)	0
Ramachandran plot	
Favored (%)	96.41
Allowed (%)	3.59
Ouliers (%)	0

Supplementary Table 1. Cryo EM data collection, refinement and validation statistics

Domain 1	Domain 2	Buried surface area (A^2)
STAS-monomer A (505-726)	STAS-monomer B (505-726)	1,192 (596+596)
STAS-monomer A (505-726)	N-terminus monomer A (13-75)	759 (353+406)
STAS-monomer B (505-726)	N-terminus monomer A (13-75)	1,438 (717+721)
STAS-monomer A (505-726)	TM-monomer A (76-504)	501(237+264)
STAS-monomer B (505-726)	TM-monomer A (76-504)	1,293 (654+630)
N-terminus monomer A (13-75)	N-terminus monomer B (13-75)	1,046 (523+523)
TM-monomer A (76-504)	TM-monomer B (76-504)	744 (372+372)
Protomer 1 (13-726)	Protomer 2 (13-726)	7,712 (3,856+3,856)

Supplementary Table 2. The combined buried surface area in several molecular interfaces observed in the cryo-EM structure of prestin from gerbil. As is evident the greatest interface is between the STAS domain of one protomer and the N-terminus of the other, followed by the STAS domain of one protomer and the TM domain of the other, the STAS domains of the two protomers, and the N-termini of the two protomers. Interface between the two TM domains has a smaller contribution.
