# Supplementary material for: Single particle cryo-EM structure of the outer hair cell motor protein prestin
Source: Nat Commun. 2022 Jan 12;13:290. doi: 10.1038/s41467-021-27915-z (PMC8755724; doi:10.1038/s41467-021-27915-z)
Supplement: Supplementary file 3 — Reporting Summary [file 41467_2021_27915_MOESM3_ESM.pdf]

nature portfolio

Corresponding author(s): D Navaratnam

Last updated by author(s): Nov 30, 2021

Reporting Summary

Nature Portfolio wishes to improve the reproducibility of the work that we publish. This form provides structure for consistency and transparency in reporting. For further information on Nature Portfolio policies, see our Editorial Policies and the Editorial Policy Checklist.

Statistics

For all statistical analyses, confirm that the following items are present in the figure legend, table legend, main text, or Methods section.

n/a	Confirmed	
☐	☒	The exact sample size (*n*) for each experimental group/condition, given as a discrete number and unit of measurement
☐	☒	A statement on whether measurements were taken from distinct samples or whether the same sample was measured repeatedly
☐	☒	The statistical test(s) used AND whether they are one- or two-sided<br>*Only common tests should be described solely by name; describe more complex techniques in the Methods section.*
☒	☐	A description of all covariates tested
☒	☐	A description of any assumptions or corrections, such as tests of normality and adjustment for multiple comparisons
☐	☒	A full description of the statistical parameters including central tendency (e.g. means) or other basic estimates (e.g. regression coefficient) AND variation (e.g. standard deviation) or associated estimates of uncertainty (e.g. confidence intervals)
☐	☒	For null hypothesis testing, the test statistic (e.g. *F*, *t*, *r*) with confidence intervals, effect sizes, degrees of freedom and *P* value noted<br>*Give P values as exact values whenever suitable.*
☒	☐	For Bayesian analysis, information on the choice of priors and Markov chain Monte Carlo settings
☒	☐	For hierarchical and complex designs, identification of the appropriate level for tests and full reporting of outcomes
☒	☐	Estimates of effect sizes (e.g. Cohen's *d*, Pearson's *r*), indicating how they were calculated

Our web collection on statistics for biologists contains articles on many of the points above.

Software and code

Policy information about availability of computer code

Data collection	Serial EM v3.8; JClamp v 31.7.0
Data analysis	Relion 3.1, Phenix 1.19.2; CTFFind4.1; Coot 0.8.9.1; MotionCor2v1.3.2; Chimera v1.12; ChimeraX v0.9; JClamp v 31.7.0

For manuscripts utilizing custom algorithms or software that are central to the research but not yet described in published literature, software must be made available to editors and reviewers. We strongly encourage code deposition in a community repository (e.g. GitHub). See the Nature Portfolio guidelines for submitting code & software for further information.

Data

Policy information about availability of data

All manuscripts must include a data availability statement. This statement should provide the following information, where applicable:

- Accession codes, unique identifiers, or web links for publicly available datasets
- A description of any restrictions on data availability
- For clinical datasets or third party data, please ensure that the statement adheres to our policy

The datasets generated during and/or analy sed during the current study will be available in the PDB repository PDB ID: 7SUN. Other data are available in the source data file.

Field-specific reporting

Please select the one below that is the best fit for your research. If you are not sure, read the appropriate sections before making your selection.

☒ Life sciences ☐ Behavioural & social sciences ☐ Ecological, evolutionary & environmental sciences

For a reference copy of the document with all sections, see nature.com/documents/nr-reporting-summary-flat.pdf

Life sciences study design

All studies must disclose on these points even when the disclosure is negative.

Sample size	Over 1 million particles were averaged for cryoEM. Purifications were repeated over 5 times. Grids of the highest quality were collected. Sample sizes were not calculated for structural data. For ephys experiments where p was > 0.05 in our paper, the absolute differences are very small and in the normal range of NLC parameters. Increasing sample size may improve the SE but will not change the absolute difference or alter our conclusion. No statistical group comparison was done in other experiments
Data exclusions	For CryoEM particles were excluded based on 2D and 3D classification. For electrophysiological experiments data were excluded where Rm values were less than 300MOhm and Rs greater than 10 MOhm as measures of NLC are unreliable.
Replication	CryoEm data were collected on more than 5 separate purifications and found to be reproducible. Grids with highest quality were used for collection. Electrophysiological experiments were repeated multiple times. Typically 5-10 recordings of separate cells expressing specific constructs of prestin or its mutants were used for analysis. All attempts at replication were successful.
Randomization	Randomization was not relevant to the structural data. Ephys experiments were done on batches of transfected cells. Randomization is done to eliminate the bias from confounding variables which distribute unbalanced between comparison groups. We compared NLC parameters on cultured cells rather than human or animal subjects. Randomization is not applicable to our study.
Blinding	Investigators were not blinded to the data. Often the same experimenter performed transfection and recordings. Where these were separate the recorder was blind to the constructs in the cells recorded. Blinding is effective to avoid artificial group difference that is caused by performance bias. We reported mutants show similar z. Unblinding has no bias on null results.

Reporting for specific materials, systems and methods

We require information from authors about some types of materials, experimental systems and methods used in many studies. Here, indicate whether each material, system or method listed is relevant to your study. If you are not sure if a list item applies to your research, read the appropriate section before selecting a response.

Materials & experimental systems

n/a	Involved in the study	
☒	☐	Antibodies
☐	☒	Eukaryotic cell lines
☒	☐	Palaeontology and archaeology
☒	☐	Animals and other organisms
☒	☐	Human research participants
☒	☐	Clinical data
☒	☐	Dual use research of concern

Methods

n/a	Involved in the study	
☒	☐	ChIP-seq
☒	☐	Flow cytometry
☒	☐	MRI-based neuroimaging

Eukaryotic cell lines

Policy information about cell lines

Cell line source(s)	ATCC. HEK cells. CHO cells.
Authentication	Cell lines were not authenticated. Prestin-YFP expression determined by fluorescence, electrophysiology and western blotting
Mycoplasma contamination	Testing not done
Commonly misidentified lines (See ICLAC register)	No commonly misidentified cell lines were used in the study
